# Beyond the physical risk: Psychosocial impact and coping in healthcare professionals during the COVID‐19 pandemic

**DOI:** 10.1111/jocn.15938

**Published:** 2021-07-06

**Authors:** Theodora Fteropoulli, Theano V. Kalavana, Anneza Yiallourou, Marios Karaiskakis, Maria Koliou Mazeri, Stavros Vryonides, Anna Hadjioannou, Georgios K. Nikolopoulos

**Affiliations:** ^1^ Medical School University of Cyprus Nicosia Cyprus; ^2^ Health Services Research, City University of London London United Kingdom; ^3^ MindThreading Nicosia Cyprus; ^4^ Breast Unit Nicosia General Hospital Nicosia Cyprus; ^5^ University of Nicosia Medical School Nicosia Cyprus; ^6^ General Surgery Ygia Polyclinic Limassol Cyprus; ^7^ Pediatric Department Archbishop Makarios Hospital Nicosia Cyprus; ^8^ Limassol General Hospital Limassol Cyprus; ^9^ State Health Services Organization Nicosia Cyprus

**Keywords:** COVID‐19, healthcare professionals, quality of life, anxiety, depression, occupational burnout, coping, SARS‐CoV‐2, Pandemic, mental health

## Abstract

**Aims and objectives:**

This study aimed to examine the psychosocial impact and identify risk factors for poor psychosocial outcomes in healthcare professionals during the Coronavirus disease 2019 (COVID‐19) pandemic in Cyprus.

**Background:**

Healthcare professionals are in the forefront of the COVID‐19 pandemic facing an unprecedented global health crisis, which can have consequences on their psychosocial health. There is a need to identify risk factors for poor psychosocial outcomes to inform the design of tailored psychological interventions.

**Design:**

Cross‐sectional online study.

**Methods:**

A total of 1071 healthcare professionals completed self‐report questionnaires. Measures included sociodemographic information, COVID‐19‐related characteristics, quality of life (Brief World Health Organization Quality of Life; WHOQOL‐Bref), anxiety (Generalized Anxiety Disorder‐7; GAD‐7), depression (Patient Health Questionnaire‐8; PHQ‐8), occupational burnout (Copenhagen Burnout Inventory; CBI), and coping (Brief Coping Orientation to Problems Experienced; Brief COPE). This article follows the STROBE reporting guidelines.

**Results:**

The prevalence of moderate to severe anxiety and clinically significant depression was 27.6% and 26.8%, respectively. Significant risk factors for poor psychological outcomes included being female, being a nurse or doctor (vs non‐medical professional), working in frontline units (inpatient, intensive care), perceptions of inadequate workplace preparation to deal with the pandemic, and using avoidance coping. Depression and occupational burnout were significant risk factors for poor quality of life.

**Conclusion:**

The findings suggest several individual, psychosocial, and organisational risk factors for the adverse psychological outcomes observed in healthcare professionals during the COVID‐19 pandemic.

**Relevance to clinical practice:**

This study highlights the urgent need for screening for anxiety and depression and psychological interventions to combat an imminent mental health crisis in healthcare professionals during the COVID‐19 pandemic. Pandemic response protocols and public health initiatives aiming to improve and prevent mental health problems in healthcare professionals during the current and future health crises, need to account for the various factors at play.

**What does this paper contribute to the wider global clinical community?**

- There are notable levels of anxiety and clinically significant depression in healthcare professionals during the COVID‐19 pandemic.
- Risk factors for poor QoL, anxiety, depression, and occupational burnout include female gender, being a nurse or doctor (vs non‐medical professional), working in frontline units (inpatient wards, ICUs), inadequate workplace preparation to deal with the pandemic, and engaging in avoidance coping, while the presence of depression and occupational burnout further impedes QoL in healthcare professionals.
- The findings can help identify healthcare professionals in need for psychological support and in the design of tailored psychological interventions, public health initiatives, and future protocols to help healthcare professionals cope with the psychosocial burden of COVID‐19 and build resilience against future health crises.

## 1 INTRODUCTION

The coronavirus disease 2019 (COVID‐19) outbreak caused by severe acute respiratory syndrome‐coronavirus‐2 (SARS‐CoV‐2) emerged in December 2019. According to the World Health Organization (WHO, 2021), there have been over 119 million confirmed cases and over 2.5 million deaths from COVID‐19 worldwide as of March 16, 2021. The Republic of Cyprus is facing an unprecedented situation like many other countries around the world, with 36,786 cases of COVID‐19 and 236 deaths until March 9, 2021 (Epidemiological Surveillance Unit of the Ministry of Health, 2021). During the early phase of the pandemic in March 2020, Cyprus was still implementing its newly formed General Healthcare System (GHS), which aimed to provide universal health coverage for the whole population. Prior to 2019, approximately 17% of Cypriots had to pay to access the public health system and/or services in the private health sector (Theodorou et al., 2012). The COVID‐19 pandemic further complicated this transitional period, causing delays in the introduction of certain healthcare services and exacerbating the shortage of healthcare staff in public hospitals (WHO Regional Office for Europe et al., 2020). Furthermore, organisational changes were made to cope with the pandemic emergency situation, whereby an entire hospital (out of six public hospitals) was converted into a COVID‐19 treatment centre suspending most other services, many clinics within hospitals were converted to COVID‐19 wards, healthcare professionals from various departments were assigned to the treatment of COVID‐19 patients, and non‐essential outpatient appointments and non‐emergency surgeries were postponed (Press & Information Office, 2020a). At times, the increasing infection rates among inpatients and hospital staff has led to temporary suspension of services of major public hospitals and clinics. A significant proportion (21.4%) of the 873 confirmed cases in Cyprus between March and May 2020 were healthcare professionals, of which approximately 50% were nurses (Quattrocchi et al., 2020).

The healthcare challenges posed by the COVID‐19 pandemic coupled with the organisational transition placed increased pressure on healthcare professionals; they have been in the forefront of the COVID‐19 pandemic response and many of them have not experienced a global health crisis of these proportions before. Additional stressors are introduced in an already hectic setting, including the increased infection risk, global lack of protective and medical equipment during the early stages, lack of a very effective COVID‐19 treatment (with a few treatment modalities approved) (Beigel et al., 2020; RECOVERY Collaborative Group et al., 2021; Sterne et al., 2020), extended working hours, caring for severely ill patients, and taking life and death decisions among others; all while being isolated from family and friends (Santarone et al., 2020). These working conditions have put healthcare professionals at greater risk for psychological distress, fatigue, burnout, and stigma (Arora et al., 2020; Muller et al., 2020; Paiano et al., 2020; Pappa et al., 2020). The WHO has therefore urged healthcare professionals to care for their mental health and use positive coping strategies to deal with the increased demands during this pandemic (WHO, 2020). Research on the most suitable ways to help our healthcare workforce cope with the psychological impact of the COVID‐19 pandemic has been identified as an immediate priority (Zaka et al., 2020).

## 2 BACKGROUND

During the SARS pandemic, healthcare workers experienced post‐traumatic stress, depression, and anxiety symptoms especially if they worked in high‐risk clinical settings (Wu et al., 2009). There is now a growing body of research focusing on the psychological consequences of COVID‐19 in healthcare professionals, with the majority originating in China. In addition to the physical risks, these studies have found increased levels of depression, anxiety, insomnia, and distress in healthcare professionals during the COVID‐19 pandemic (Lai et al., 2020; Xiao et al., 2020; J. Zhang et al., 2020). Several factors have been reported to increase the risk for psychological symptoms in healthcare workers, including being a nurse, female gender, and frontline work (Muller et al., 2020; Pappa et al., 2020). The increased workload and psychological distress during the COVID‐19 pandemic can further decrease work engagement in frontline nurses potentially influencing the provision of quality of care (Zhang et al., 2021). To our knowledge, there is limited evidence on the psychosocial impact of COVID‐19 on healthcare professionals in Cyprus, a country that has no extensive experience in dealing with pandemics of this scale and lacks psychological support services tailored to the needs of healthcare professionals.

It is also important to identify the underlying psychological processes, which can serve as protective factors against the negative psychosocial consequences of the pandemic in healthcare professionals. One such factor is coping, which is motivated action underlined by self‐regulatory processes that helps people adapt to stressful situations (Carver, 1997). The SARS literature has highlighted the role of maladaptive coping skills, including substance use, self‐blame, and avoidance as risk factors for psychological symptoms in healthcare professionals even long after the outbreak was resolved (Maunder et al., 2008). Healthcare professionals working in different roles and settings during the SARS epidemic used diverse maladaptive coping strategies. For example, a study in Hong Kong reported that higher levels of distress in emergency doctors dealing with SARS were associated with greater use of venting as a coping strategy, whereas higher levels of distress in nurses were associated with greater use of behavioural disengagement and self‐distraction (Wong et al., 2005). Coping and how it relates to the psychological responses and QoL of healthcare professionals during COVID‐19 have received little attention. A study conducted in Italy has found that a positive attitude held by healthcare professionals during COVID‐19 was associated with lower perceived stress, whereas seeking support and avoidance coping were associated with higher perceived stress (Babore et al., 2020). A qualitative study, on the other hand, has highlighted the benefit of having a support system in terms of stress in healthcare workers during the COVID‐19 pandemic (Eftekhar Ardebili et al., 2020).

There is need for more evidence on how the use of certain maladaptive coping strategies may put healthcare professionals at risk for mental health problems and poor QoL that will help inform a strategy to enhance their coping skills and support them during this pandemic. Such research will satisfy the urgent need, as highlighted by a recent call for action paper, to provide psychological interventions for healthcare professionals tailored to their current needs (Zaka et al., 2020). The focus of much of the research has been on anxiety and depression as psychological outcomes. Quality of Life (QoL) is an equally important outcome as it captures various aspects of a person's life, including physical health, psychological well‐being, environment, and work. QoL challenges in healthcare professionals may persist long after the current outbreak subsides and even if anxiety and depression are not present.

The present study sought to address these research gaps and contribute to the growing literature by examining the psychosocial impact on healthcare professionals and their coping skills during the COVID‐19 pandemic in Cyprus, identifying risk factors for poor psychosocial outcomes. The objectives were: (a) to examine the impact of sociodemographic and COVID‐19‐related characteristics and coping skills on the psychosocial functioning (anxiety, depression, occupational burnout) and QoL of healthcare professionals during the COVID‐19 pandemic and (b) to investigate differences in the use of coping skills among healthcare professionals working in different contexts and settings.

## 3 METHODS

### 3.1 Design

We conducted a cross‐sectional study to examine the impact of various factors on the psychosocial functioning and QoL of healthcare professionals during the COVID‐19 pandemic in Cyprus. The present study adhered to the Strengthening the Reporting of Observational Studies in Epidemiology (STROBE) guidelines for observational research—cross‐sectional studies (Vandenbroucke et al., 2007) (see Supplementary File 1).

### 3.2 Setting

The study consisted of an anonymous online survey administered between May 25th and October 27th, 2020 in Cyprus.

### 3.3 Participants

The study followed a convenience sampling approach and included healthcare professionals of the public and private sector defined as physicians, nurses, allied health professionals, and health scientists and academics involved in the Cypriot government's efforts to combat COVID‐19. Exclusion criteria included: (a) healthcare professionals who were not working or were absent (e.g., abroad, medical leave, sabbatical leave) during the whole duration of the pandemic, (b) inability to consent without the help of a third party, (c) inability to understand Greek. Participants were recruited through mass e‐mails from professional organisations, including the Cyprus Medical Association (CYMA) and the Cyprus Nurses and Midwives Association (CYNMA), social media platforms, and national newspapers. Prospective participants were provided with a study link containing information about the study, the electronic consent form, and the survey. The survey was administered through the Research Electronic Data Capture (REDCap) (Harris et al., 2009) platform hosted at the University of Cyprus and took approximately 15 min to complete.

### 3.4 Measures

The survey consisted of three sections of self‐report questionnaires. The first section included sociodemographic and COVID‐19‐related characteristics, including age, gender, family status, education, profession, years of experience in clinical settings, experience in crisis situations in clinical settings, work setting and department during the pandemic, contact with COVID‐19 patients (frontline vs non‐frontline staff), adequacy of COVID‐19 preparation in their workplace, need for self‐isolation during the pandemic, and whether they were diagnosed with COVID‐19. The second section included questionnaires relating to psychosocial functioning and QoL. QoL was assessed using the Greek translation of the World Health Organization Quality of Life measure (WHO‐QOL BREF) (Ginieri‐Coccossis et al., 2012). Participants are asked to rate their QoL and health status in the past two weeks using five‐point Likert scales. Scores are obtained in four domains: physical health (nine items), psychological health (six items), social relationships (five items), and environment (eight items), and an overall QoL score. The mean scores in each domain are multiplied by four and can range between 4 and 20, with higher scores indicating better QoL. Anxiety was measured using the Greek translation of the Generalized Anxiety Disorder‐7 (GAD‐7) (Spitzer et al., 2006). Participants are asked to rate how often they were bothered by certain issues during the past two weeks using a three‐point Likert scale of seven items. The total score can range from zero to 21 with higher scores indicating higher anxiety levels. Scores of 5, 10, and 15 may represent cut‐off points for mild, moderate, and severe anxiety, respectively (Spitzer et al., 2006). Depression was measured with the Greek translation of the Patient Health Questionnare‐8 (PHQ‐8) (Kroenke et al., 2001, 2009). Participants are asked to rate how often they were bothered by certain issues during the past two weeks using a three‐point Likert scale of eight items. The total score can range from zero to 24 with higher scores indicating higher depression levels. A total score of zero to 4 represents no significant depressive symptoms, 5 to 9 represents mild symptoms, 10 to 14 represents moderate symptoms, 15 to 19 represents moderately severe symptoms, and 20 to 24 represents severe symptoms. A cut‐off point of larger or equal to ten defines current depression (Kroenke et al., 2001, 2009). Occupational burnout was assessed using the relevant scale of the Greek translation of the Copenhagen Burnout Inventory (CBI) (Kristensen et al., 2005; Papaefstathiou et al., 2019). Participants are asked to rate the degree to which they experience exhaustion due to their work using a five‐point Likert scale of seven items. The mean score can range between 0 and 100, with higher scores indicating greater occupational burnout. The third section included the Greek translation of the Brief Coping Orientation to Problems Experienced inventory (Brief COPE) (Carver, 1997) which assesses individuals’ coping strategies as self‐regulatory processes. Participants are asked to indicate the degree to which they used each strategy when they experienced stress during the past two weeks using a four‐point Likert scale. The instrument measures 14 coping reactions (two items each), including active coping, planning, positive reframing, acceptance, humour, religion, using emotional support, using instrumental support, self‐distraction, denial, venting, substance use, behavioural disengagement, and self‐blame. Mean scores are obtained for each subscale which can range between 0 and 3, where higher scores indicate greater use of the specific coping strategy. Exploratory factor analysis may be performed on the subscales of the Brief COPE to explore the underlying second‐order factors (Carver, 1997).

### 3.5 Statistical analyses

To estimate a statistically sufficient sample size for the study, *a priori* power analysis using G‐Power (Faul et al., 2007) was conducted. The sample size was calculated with the aim of detecting a medium effect size (Cohen, 1988) in the main outcomes (quality of life, anxiety, depression, burnout) and at *α* = 0.05. Approximately 179 participants would be needed to achieve a power of 90% when using linear multiple regression analysis with a maximum of 17 predictor variables. These calculations were used as a reference point to indicate the minimum number of participants required for a statistically sufficient sample size and there was no maximum threshold.

Data from REDCap were exported to IBM SPSS 26, where all analyses were performed. Details of the missing value analysis are provided in Supplementary File 2. Complete case analysis was selected since missingness was associated with independent variables, producing no great risk for bias (Hughes et al., 2019). Exploratory factor analysis (EFA) was conducted on the BRIEF COPE subscales using Principal Axis Factoring extraction with orthogonal (varimax) rotation to identify second‐order factors. The appropriateness of EFA was confirmed by examining the factorability, the Kaiser–Meyer–Olkin measure of sampling adequacy, Bartlett's test of sphericity, and the values of the diagonals of the anti‐image correlation matrix. Factors were extracted based on the Kaiser rule (eigenvalues > 1) and the scree plot. A cut‐off of >0.4 was selected as the minimum factor loading (Field, 2009). Composite scale scores were calculated based on the mean score of the subscales that loaded onto a given factor. Descriptive analyses were performed for sociodemographic, COVID‐19‐related variables, anxiety, and depression. Multiple linear regressions were performed to examine the demographic, COVID‐19‐related, and psychosocial predictors of anxiety, depression, occupational burnout, and QoL outcomes. In the regression models for QoL outcomes, anxiety, depression, and burnout were also entered as predictors. Predictors were entered in the models using the entry method and if they were significantly associated with the outcomes in bivariate correlations. Dummy variables were used where appropriate. To examine differences in the use of coping skills among professionals working in different contexts and settings, we conducted independent samples t‐tests and one‐way ANOVA with post hoc tests (Tukey HSD for equal or Games–Howell for unequal variances) based on 95% confidence intervals. The Welch test was performed when the homogeneity of variances assumption was violated. A *p*‐value < .05 (two‐tailed) was considered to indicate statistical significance.

### 3.6 Ethical statement

The study was conducted according to the guidelines of the Declaration of Helsinki and has received ethical approval from the Cyprus National Bioethics Committee (approval number: 2020.01.106). Participants completed an online consent form and all data collected were anonymous and confidential.

## 4 RESULTS

Results of missing value analysis and differences between complete and incomplete cases are available in Supplementary File 2. The total number of participants was 1071 and 73% were female, while mean age was 36.86 (±8.93). Detailed sociodemographic and COVID‐19‐related characteristics and prevalence of anxiety and depression are presented in Table 1. Healthcare professionals who experienced some level of anxiety and depression were 748 (69.9%) and 715 (66.9%), respectively. Of those, 292 (27.6%) experienced at least moderate anxiety levels, while 286 (26.8%) potentially had current and clinically significant depression.

**TABLE 1:** Sociodemographic, COVID‐19‐related characteristics, and prevalence of anxiety and depression in the sample (*n *= 1071)

Characteristic		Grouping	*n*	%	Mean (S.D.)	Range
Age					36.86 (8.93)	21–69
Gender	Female		782	73		
	Male		289	27		
Family status†	Married/in a relationship		783	73.1		
	Single		288	26.9		
Education level‡	University level		1060	99		
	School level		11	1		
Profession§	Doctors		39	3.6		
	Nurses & midwives		974	90.9		
	Non‐medical professionals		58	5.4		
Years of experience					13.30 (8.47)	0–46
Healthcare setting¶	Primary		80	7.5		
	Outpatient		47	4.4		
	Emergency		111	10.4		
	Inpatient		511	47.7		
	Intensive care unit		138	12.9		
	Mental health		41	3.8		
	Specialised		60	5.6		
	Lab		34	3.2		
	Public health		49	4.6		
Healthcare crisis experience	No		903	84.3		
	Yes		168	15.7		
Frontline	No		583	54.4		
	Yes		488	45.6		
COVID−19 preparation	No		465	43.4		
	Yes		606	56.6		
Self‐isolation	No		703	65.6		
	Yes		368	34.4		
COVID−19 diagnosis	No		1046	97.7		
	Yes		25	2.3		
Anxiety	No/minimal		323	30.2		
	Mild		456	42.6		
	Moderate		199	18.6		
	Severe		93	8.7		
Depression	No/minimal		356	33.2		
	Mild		429	40.1		
	Moderate¥		188	17.6		
	Moderately severe¥		82	7.7		
	Severe¥		16	1.5		

^†^ Single, divorced/separated, and widowed were merged into the ‘single’ category

^‡^ Primary, secondary, and high school were merged into the ‘school level’ category, whereas university degree, postgraduate degree, and doctorate were merged into the ‘university level’ category

^§^ Allied health professionals, administrative, academic, and support staff were merged into the ‘non‐medical professionals’ category

^¶^ This variable was created based on participants’ self‐reported work setting and department

^¥^ Scores that indicate current and clinically significant depression.

### 4.1 Exploratory factor analysis of the Brief COPE

EFA of the Brief COPE yielded a three‐factor solution based on eigenvalues >1, which explained 41.65% of the variance. The scree plot (see Supplementary File 3) confirmed the three‐factor structure. The subscales of humour, religion, and substance use did not load on any of the factors at >0.40 and had low communalities (<0.20), thus were removed from the model. This resulted in a better three‐factor solution, which explained 48.92% of the variance. Table 2 contains the results of EFA with the rotated factor loadings. The three extracted factors represented ‘Approach’ (active efforts to deal with the problem), ‘Support‐seeking’ (seeking support from the environment), and ‘Avoidance’ (avoiding dealing with the problem) coping.

**TABLE 2:** Exploratory factor analysis of the Brief COPE (*n* = 1071)

	Rotated Factor Loadings		
Subscales	Factor 1 Approach Coping	Factor 2 Support‐seeking Coping	Factor 3 Avoidance Coping
Planning	0.78	0.19	0.02
Positive reframing	0.71	0.17	−0.12
Active Coping	0.60	0.24	0.15
Acceptance	0.52	0.09	−0.06
Self‐blame	0.46	0.16	0.35
Self‐distraction	0.43	0.21	0.38
Instrumental support	0.21	0.85	0.12
Emotional support	0.20	0.78	0.16
Venting	0.33	0.44	0.22
Denial	0.10	0.13	0.71
Behavioural disengagement	−0.18	0.10	0.59
Eigenvalues	2.37	1.76	1.25
% of variance	21.57	15.97	11.37
Cumulative %	21.57	37.55	48.92

Notes

Kaiser‐Meyer‐Olkin (KMO) = 0.80; Bartlett's test of sphericity: *χ* ^2^(55) = 381419, *p* < .001; Factor loading threshold set at >0.4.

### 4.2 Predictors of anxiety, depression, and occupational burnout

The correlations between variables that informed the multiple linear regressions are available in Supplementary File 4. The coefficients of multiple regressions for anxiety, depression, and occupational burnout are presented in Table 3. Considering the sociodemographic predictors, being female was associated with higher levels of anxiety and occupational burnout (beta = 0.07, *p* < .05), being a medical doctor (vs non‐medical professional) was associated with greater occupational burnout (beta = 0.08, *p* < .05), while being a nurse or midwife (vs non‐medical professional) was associated with greater depression and occupational burnout (betas = 0.08 and 0.10, *p* < .05). Working in inpatient and Intensive Care Unit (ICU) settings (vs public health setting) was associated with greater occupational burnout (betas = 0.16 and 0.11, *p* < .05), while working in a mental health setting (vs public health setting) was associated with lower anxiety (beta = −0.08, *p* < .05). Working in the frontline was associated with greater occupational burnout (beta = 0.07, *p* < .05) and inadequate workplace preparation to deal with the pandemic was associated with greater anxiety (beta = −.06, *p *< .05), depression (beta = −.06, *p *< .05), and occupational burnout (beta = −.22, *p *< .001). Greater use of avoidance coping was also associated with worse scores in anxiety (beta = 0.44, *p *< .001), depression (beta = .48, *p *< .001), and occupational burnout (beta = .27, *p *< .001).

**TABLE 3:** Multiple linear regressions for anxiety, depression, and occupational burnout (*n* = 1071)

	Anxiety				Depression				Occupational burnout			
	*B (SE)*	*β*	*95% CI*	*p*	*B (SE)*	*β*	*95% CI*	*p*	*B (SE)*	*β*	*95% CI*	*p*
Gender†	.74 (.29)	.07	.18, 1.31	.010	‐	‐			24.63 (9.71)	.07	5.58, 43.69	.011
Family status‡	.51 (.28)	.05	−.04, 1.07	.069	‐	‐			‐	‐		
Doctor§	‐	‐			.83 (.89)	.03	−.91, 2.56	.352	64.26 (28.88)	.08	7.59, 120.93	.026
Nurses & midwives§	‐	‐			1.26 (.58)	.08	.14, 2.39	.028	52.40 (18.70)	.10	15.70, 89.09	.005
Primary¶	−.01 (.74)	−.00	−1.48, 1.45	.985	.55 (.77)	.03	−.96, 2.07	.473	−41.25 (25.22)	−.07	−90.74, 8.23	.102
Outpatient¶	−1.53 (.83)	−.07	−3.16,.11	.067	−1.13 (.87)	−.05	−2.83,.57	.191	−3.19 (28.22)	−.00	−58.57, 52.19	.910
Emergency¶	−.09 (.71)	−.01	−1.48, 1.30	.903	−.25 (.73)	−.02	−1.67, 1.18	.732	27.07 (24.09)	.06	−20.20, 74.33	.261
Inpatient¶	.12 (.61)	.01	−1.08, 1.32	.842	.35 (.63)	.04	−.89, 1.59	.578	47.21 (20.60)	.16	6.79, 87.63	.022
Intensive care unit¶	1 (.68)	.01	−1.24, 1.43	.889	.35 (.70)	.02	−1.02, 1.72	.618	51.17 (23.25)	.11	5.56, 96.78	.028
Mental health¶	−1.84 (.87)	−.08	−3.56, −.13	.035	−1.47 (.89)	−.06	−3.21,.28	.100	31.33 (29.24)	.04	−26.05, 88.71	.284
Specialised¶	.02 (.79)	.00	−1.52, 1.57	.977	.80 (.81)	.04	−.78, 2.39	.321	32.95 (26.38)	.05	−18.82, 84.72	.212
Lab¶	−.17 (.91)	−.01	−1.96, 1.62	.850	.70 (.94)	.03	−1.15, 2.54	.458	36.42 (30.66)	.04	−23.75, 96.59	.235
Frontline¥	‐	‐			‐	‐			21.13 (9.21)	.07	3.06, 39.19	.022
Preparation¥	−.56 (.26)	−.06	−1.06, −.06	.028	−.56 (.26)	−.06	−1.07, −.04	.035	−65.27 (8.57)	−.22	−82.09, −48.45	<.001
Self‐isolation¥	‐	‐			‐	‐			6.03 (9.15)	.02	−11.93, 23.98	.510
Approach coping	−.26 (.27)	−.03	−.79,.27	.332	‐	‐			‐	‐		
Support‐seeking coping	.42 (.23)	.06	−.03,.86	.065	−.01 (.20)	−.00	−.41,.38	.944	−7.36 (6.65)	−.03	−20.40, 5.69	.269
Avoidance coping	3.71 (.24)	.44	3.25, 4.18	<.001	4.16 (.24)	.48	3.68, 4.64	<.001	71.96 (7.92)	.27	56.42, 87.50	<.001
*R^2^*	.24				.25				.19			
Adjusted *R^2^*	.23				.24				.18			
*f* test	*f*(14, 1056) = 23.90, *p* < .001				*f*(13, 1057) = 26.98, *p* < .001				*f*(16, 1054) = 15.33, *p* < .001			

Note

‐ indicates that the variable was not part of the multiple regression model; B indicates unstandardised coefficient, *β* indicates standardised coefficient.

^†^ Male = 1, female = 2.

^‡^ Single = 0, Married/in a relationship = 1.

^§^ Dummy‐coded: reference group was non‐medical profession.

^¶^ Dummy‐coded: reference group was public health setting.

^¥^ No = 0, Yes = 1.

### 4.3 Predictors of quality of life

The coefficients of multiple regressions for QoL outcomes are presented in Table 4. Being female was associated with poorer psychological health (beta = −0.07, *p *< .05), older age was associated with poorer overall health (beta = −0.26, *p *< .001), and being single and being a medical doctor (vs non‐medical professional) were associated with poorer social relationships (beta = 0.14, *p *< .001 and beta = −0.09, *p *< .05, respectively). Two COVID‐19‐related predictors were significantly associated with QoL outcomes. Inadequate workplace preparation to deal with the pandemic was associated with poorer overall QoL (beta = 0.07, *p *< .05), social relationships (beta = 0.07, *p *< .05), and environmental QoL (beta = 0.10, *p *< .001), while being diagnosed with COVID‐19 was associated with poorer environmental QoL (beta = −0.06, *p *< .05). The single most common psychosocial predictor of poor QoL across all outcomes was depression (betas = −0.29 to −0.52, *p *< .001). Occupational burnout was associated with poor QoL in all outcomes except psychological health (betas = −0.11 to −0.19, *p *< .001). Anxiety was only associated with poor environmental QoL (beta = −0.10, *p *< .05). Using avoidance coping in greater extent was associated with poorer psychological health (beta = −0.14, *p *< .001) and social relationships (beta = −0.07, *p* < .05).

**TABLE 4:** Multiple linear regressions for QoL outcomes (*n* = 1071)

	Overall Health/QoL			Physical Health			Psychological Health			Social Relationships			Environment		
	*B (SE)*	*β*	*95% CI*	*B (SE)*	*β*	*95% CI*	*B (SE)*	*β*	*95% CI*	*B (SE)*	*β*	*95% CI*	*B (SE)*	*β*	*95% CI*
Gender†	‐	‐		−.17 (.11)	−.04	−.38,.04	−.35 (.11)	−.07**	−.57, −.12	‐	‐		‐	‐	
Age	−.07 (.02)	−.26***	−.11, −.03	‐	‐		‐	‐		‐	‐		‐	‐	
Family status‡	‐	‐		‐	‐		‐	‐		.75 (.13)	.14***	.49, 1.01	‐	‐	
Doctor§	‐	‐		.09 (.32)	.01	−.54,.72	‐	‐		−1.09 (.40)	−.09**	−1.87, −.32	‐	‐	
Nurses & midwives§	‐	‐		.03 (.21)	.00	−.38,.43	‐	‐		.04 (.26)	.01	−.47,.55	‐	‐	
Years of experience	.04 (.02)	.13	−.00,.08	‐	‐		‐	‐		‐	‐		‐	‐	
Primary¶	.55 (.40)	.06	−.25, 1.34	−.17 (.28)	−.02	−.72,.38	‐	‐		‐	‐		‐	‐	
Outpatient¶	−.15 (.45)	−.01	−1.04,.73	−.20 (.31)	−.02	−.82,.41	‐	‐		‐	‐		‐	‐	
Emergency¶	.07 (.38)	.01	−.68,.82	−.07 (.26)	−.01	−.59,.44	‐	‐		‐	‐		‐	‐	
Inpatient¶	.32 (.34)	.06	−.34,.97	−.12 (.23)	−.03	−.57,.32	‐	‐		‐	‐		‐	‐	
Intensive care unit¶	.29 (.37)	.04	−.44, 1.02	.11 (.25)	.02	−.39,.61	‐	‐		‐	‐		‐	‐	
Mental health¶	−.36 (.47)	−.03	−1.28,.57	−.42 (.33)	−.04	−1.06,.21	‐	‐		‐	‐		‐	‐	
Specialised¶	.19 (.43)	.02	−.65, 1.03	−.17 (.29)	−.02	−.74,.41	‐	‐		‐	‐		‐	‐	
Lab¶	.01 (.49)	.00	−.95,.98	−.13 (.34)	−.01	−.80,.53	‐	‐		‐	‐		‐	‐	
Preparation¥	.36 (.14)	.07*	.08,.63	.18 (.10)	.04	−.01,.37	.13 (.10)	.03	−.08,.34	.32 (.12)	.07**	.08,.56	.37 (.10)	.10***	.17,.58
COVID−19 diagnosis¥	‐	‐		‐	‐		‐	‐		‐	‐		−.68 (.33)	−.06*	−1.32, −.03
Anxiety	.02 (.02)	.04	−.03,.07	−.02 (.02)	−.04	−.05,.01	−.03 (.02)	−.06	−.06,.01	−.03 (.02)	−.05	−.07,.02	−.04 (.02)	−.10*	−.07, −.01
Depression	−.18 (.02)	−.36***	−.23, −.14	−.22 (.02)	−.52***	−.25, −.19	−.24 (.02)	−.52***	−.28, −.21	−.23 (.02)	−.45***	−.27, −.19	−.11 (.02)	−.29***	−.14, −.08
Occupational burnout	−.00 (.00)	−.12***	−.00, −.00	−.00 (.00)	−.19***	−.00, −.00	−.00 (.00)	−.05	−.00,.00	−.00 (.00)	−.11***	−.00, −.00	−.00 (.00)	−.17***	−.00, −.00
Approach coping	.00 (.15)	.00	−.29,.30	‐	‐		‐	‐		‐	‐		‐	‐	
Support‐seeking coping	−.10 (.12)	−.03	−.34,.14	.10 (.07)	.03	−.04,.25	.15 (.08)	.05	−.00,.31	‐	‐		‐	‐	
Avoidance coping	−.12 (.15)	−.03	−.40,.17	−.01 (.10)	−.00	−.21,.19	−.57 (.11)	−.14***	−.78, −.36	−.31 (.12)	−.07**	−.55, −.07	.13 (.10)	.04	−.08,.33
*R^2^*	.22			.47			.46			.38			.25		
Adjusted *R^2^*	.20			.46			.46			.38			.25		
*f* test	*f*(17, 1053) = 17.02, *p* < .001			*f*(17, 1053) = 53.95, *p* < .001			*f*(7, 1063) = 131.36, *p* < .001			*f*(8, 1062) = 81.71, *p* < .001			*f*(6, 1064) = 58.85, *p* < .001		

Note

‐ indicates that the variable was not part of the multiple regression model; B indicates unstandardised coefficient, β indicates standardised coefficient.

^†^ Male = 1, female = 2.

^‡^ Single = 0, Married/in a relationship = 1.

^§^ Dummy‐coded: reference group was non‐medical profession.

^¶^ Dummy‐coded: reference group was public health setting.

^¥^ No = 0, Yes = 1.

* *p* ≤ .05.

** *p* ≤ .01.

*** *p* ≤ .001.

A summary of the statistically significant predictors across all psychosocial outcomes is provided in Supplementary File 5.

### 4.4 Differences in the use of coping skills

The results of comparisons in the use of coping skills based on sociodemographic and COVID‐19‐related characteristics are available in Table 5. Women used approach and support‐seeking coping to a greater extent than men (mean difference = −0.11, *p *= .002, 95%CI [−0.18, −0.04] and mean difference = −0.21, *p *< .001, 95%CI [−0.30, −0.12], respectively). Professionals working in inpatient, mental health, and public health settings used support‐seeking to a greater extent compared with those working in emergency care settings (mean difference = 0.25, *p* = .008, 95%CI [0.04,0.47], mean difference = 0.41, *p* = .020, 95%CI [0.04,0.78], and mean difference = 0.48, *p* = .001, 95%CI [0.13,0.83], respectively). Further, those working in public health settings used support‐seeking to a greater extent compared with those working in primary care settings (mean difference = 0.39, *p* = .034, 95%CI [0.02,0.76]). Professionals working in inpatient, ICU, and public health settings used avoidance coping to a greater extent compared with those working in primary care settings (mean difference = 0.21, *p* = .005, 95%CI [0.04,0.38], mean difference = 0.26, *p* = 0.007, 95%CI [0.04,0.47], and mean difference = 0.34, *p* = .020, 95%CI [0.03,0.64], respectively). Professionals who believed that their workplace was inadequately prepared to deal with the pandemic reported greater use of avoidance coping compared with those who believed that there was adequate preparation (mean difference = 0.09, *p* = .009, 95%CI [0.02,0.15]). Professionals who were diagnosed with COVID‐19 used avoidance coping to a greater extent compared with the rest (mean difference = −0.23, *p* = 0.038, 95%CI [−0.45, −0.01]).

**TABLE 5:** Mean differences in coping skills by sociodemographic and COVID‐19‐related characteristics (*n* = 1071)

Variable	Grouping	Approach Coping (Mean, S.D.)	Support‐seeking Coping (Mean, S.D.)	Avoidance Coping (Mean, S.D.)
Gender	Male	2.68 (.52)	2.15 (.65)	1.56 (.55)
	Female	2.79 (.53)	2.36 (.66)	1.61 (.55)
	*Test Statistic*	*t*(1069) = −3.07, *p *= .002	*t*(1069)= −4.66, *p* < .001	*t*(1069) = −1.26, *p*=.210
Family status	Single	2.76 (.55)	2.27 (.64)	1.59 (.56)
	Married/in a relationship	2.76 (.52)	2.32 (.67)	1.60 (.55)
	*Test Statistic*	*t*(1069) = .03, *p*=.978	*t*(1069) = −1.17, *p*=.241	*t*(1069) = −.31, *p*=.759
Education level	School level	2.80 (.52)	2.32 (.84)	1.82 (.53)
	University level	2.76 (.53)	2.31 (.66)	1.59 (.55)
	*Test Statistic*	*t*(1069) = .24, *p* = .814	*t*(1069) = .06, *p* = .949	*t*(1069) = 1.36, *p* = .175
Profession	Doctors	2.84 (.52)	2.35 (.70)	1.51 (.47)
	Nurses & midwives	2.75 (.53)	2.30 (.66)	1.60 (.56)
	Non‐medical	2.90 (.45)	2.38 (.73)	1.59 (.51)
	*Test Statistic*	*f*(2, 1068) = 2.81, *p* = .061	*f*(2, 1068)=.44, *p* = .644	*f*(2, 1068) = .45, *p* = .641
Healthcare setting	Primary	2.72 (.59)	2.19 (.74)	1.39 (.44)
	Outpatient	2.86 (.59)	2.24 (.72)	1.70 (.60)
	Emergency	2.72 (.48)	2.10 (.56)	1.57 (.54)
	Inpatient	2.75 (.52)	2.35 (.66)	1.60 (.54)
	Intensive care unit	2.75 (.57)	2.28 (.60)	1.64 (.56)
	Mental health	2.78 (.54)	2.51 (.72)	1.51 (.58)
	Specialised	2.79 (.44)	2.27 (.70)	1.56 (.54)
	Lab	2.63 (.61)	2.17 (.76)	1.73 (.68)
	Public health	2.93 (.44)	2.58 (.66)	1.72 (.58)
	*Test Statistic*	*f*(8, 1062) = 1.31, *p* = .237	*f*(8, 1062) = 3.81, *p* < .001	*f*(8, 204.61) = 3.07^a^, *p* = .003
Crisis experience	No	2.75 (.53)	2.31 (.66)	1.59 (.55)
	Yes	2.80 (.55)	2.27 (.71)	1.61 (.54)
	*Test Statistic*	*t*(1069) = −1.02, *p* = .309	*t*(1069) = .78, *p* = .437	*t*(1069) = −.35, *p* = .729
Frontline	No	2.77 (.53)	2.31 (.69)	1.59 (.56)
	Yes	2.75 (.53)	2.30 (.64)	1.59 (.55)
	*Test Statistic*	*t*(1069) = .72, *p* = .473	*t*(1069) = .31, *p* = .753	*t*(1069) = −.06, *p* = .949
Workplace preparation	No	2.79 (.52)	2.35 (.68)	1.64 (.58)
	Yes	2.73 (.54)	2.27 (.65)	1.56 (.53)
	*Test Statistic*	*t*(1069) = 1.79, *p* = .073	*t*(1069) = 1.78, *p* = .076	*t*(1069) = 2.60, *p* = .009
Self‐isolation	No	2.76 (.52)	2.31 (.67)	1.60 (.56)
	Yes	2.76 (.56)	2.30 (.65)	1.59 (.54)
	*Test Statistic*	*t*(1069) = −.14, *p*=.888	*t*(1069) = .22, *p* = .828	*t*(1069) = .26, *p* = .798
COVID−19 diagnosis	No	2.76 (.53)	2.31 (.67)	1.59 (.55)
	Yes	2.68 (.42)	2.15 (.46)	1.82 (.56)
	*Test Statistic*	*t*(1069) = .78, *p* = .438	*t*(1069) = 1.72, *p* = .227	*t*(1069) = −2.08, *p* = .038

a Welch test.

## 5 DISCUSSION

This study investigated the psychosocial impact of COVID‐19 and coping in healthcare professionals during the pandemic. It provides important indications for public health strategies focused on mental health to help reinforce healthcare professionals during the present and future health crises. Overall, there were considerable levels of clinical anxiety and depression in our sample. Several factors were found to be associated with poorer psychosocial outcomes, including female gender, medical (vs non‐medical) profession, frontline (vs non‐frontline) work, and use of avoidance coping. Depression and occupational burnout were further associated with poorer QoL outcomes.

Our findings indicated that over two thirds of healthcare professionals experienced some level of anxiety (69.9%) and depression (66.9%) and over a quarter experienced moderate to severe anxiety levels (27.6%) and potentially had clinically significant depression (26.8%). The levels of clinically significant anxiety and depression observed in this sample of healthcare professionals are higher than those reported in the general Cypriot population (23.1% and 9.2%, respectively) during the COVID‐19 pandemic (Solomou & Constantinidou, 2020). High prevalence rates of anxiety and depression have also been reported in other Mediterranean countries, including Spain (prevalence 79.3% and 51.3%, respectively) (Luceño‐Moreno et al., 2020), Italy (prevalence 71.6% and 60.3%, respectively) (Erquicia et al., 2020), and Egypt (prevalence 76.4% and 77.2%, respectively) (Elkholy et al., 2020). The rates for Chinese samples tended to be lower with reports for anxiety at 10.4%‐54.1% and depression at 10.6%‐57.3% (Xiao et al., 2020; Zhang et al., 2020). This difference likely reflects the vast experience and better preparation of Chinese healthcare professionals in dealing with epidemics compared with their Mediterranean counterparts (Zaka et al., 2020). The findings suggest that the risk for anxiety and depression in healthcare professionals has heightened during the COVID‐19 pandemic as a past systematic review reported overall rates of no more than 45% for anxiety and 27% for depression under usual circumstances (Harvey et al., 2009).

The findings also showed that being female was associated with greater anxiety and occupational burnout and poorer psychological QoL. This is in line with research from other countries (Lai et al., 2020; Luceño‐Moreno et al., 2020; Xiao et al., 2020) that have reported greater levels of depression, anxiety, and distress in female compared with male healthcare professionals. Most women were nurses or midwives in our sample. Nurses and midwives (vs non‐medical professionals) reported higher levels of occupational burnout. Recent studies attributed the high levels of burnout in nurses during the COVID‐19 pandemic to being involved in the direct care of patients as opposed to non‐medical professionals and their increased workload during the pandemic (Lasalvia et al., 2021). Despite these negative outcomes for women, they tended to use approach and support‐seeking coping to a greater extent compared with men in this study. It is generally expected that these coping styles would offer protection against stress; however, it is worth interpreting this result within the context of the COVID‐19 pandemic. Approach coping involves thinking of ways and taking active steps to deal with the stressor and ameliorate its effects, in other words trying to take control of an otherwise uncontrollable situation like the pandemic. A recent qualitative study found that a key theme in the experience of healthcare professionals during the COVID‐19 pandemic was the increased workload along with feelings of losing control (Eftekhar Ardebili et al., 2020). Support‐seeking involves seeking support from one's environment and venting of negative emotions, all of which would be constituted difficult during the pandemic due to restrictions in contacts, social distancing, and quarantine measures. This is especially relevant for female healthcare professionals in Cyprus who have an active role within their family environment, often caring for their families. Thus, as suggested by previous studies, seeking quality support by healthcare professionals during the pandemic is hindered by avoiding direct contact due to the need to protect their social circle (Muller et al., 2020). Further qualitative studies may help clarify these underlying coping processes.

Another key finding of the study was that profession and work setting was associated with psychological outcomes. Overall, our findings suggested that medical professionals (vs non‐medical), those working in inpatient and ICU settings (vs public health), and those in the frontline (vs non‐frontline) experienced greater psychological impact especially in relation to occupational burnout. Furthermore, nurses and midwives were at a higher risk for depression. These findings are in accordance with a recent meta‐analysis on the psychological impact of COVID‐19 in healthcare professionals and the role of work setting (Pappa et al., 2020). Inpatient wards and ICUs are at the centre of this pandemic hence increased workload and subsequent burnout were expected. In these units, medical professionals provide direct care to patients, experience the toll of the pandemic, and take important life‐death decisions. Our study further revealed that organisational weaknesses can negatively influence healthcare professionals. Specifically, the perception of inadequate workplace preparation was associated with multiple psychological outcomes, including worse QoL outcomes and greater anxiety, depression, and occupational burnout. Our assessment of workplace preparation consisted of a single item; thus, it is not possible to pinpoint the specific organisational weaknesses responsible. Past research has suggested that lack of personal protective equipment, insufficient training in pandemic response, limited ICU beds and respirators, long shifts, and lack of support are some organisational failings that contributed to worse psychological outcomes in healthcare professionals during the COVID‐19 pandemic (Paiano et al., 2020; Suryavanshi et al., 2020).

Our findings also showed that avoidance coping was associated with worse psychological outcomes. Avoidance coping represents strategies including denial and giving up, which have been deemed maladaptive both in the current (Babore et al., 2020) and the SARS epidemic (Wong et al., 2005). The impact of work setting and perceptions of organisational weaknesses on psychosocial outcomes discussed above may be further elucidated by the differences observed in the use of avoidance coping. Interestingly, professionals working in inpatient and ICU settings and those who perceived inadequate workplace preparation used avoidance coping in greater extent than those working in primary settings and who perceived adequate preparation, respectively. It appears that when faced with deficiencies in terms of preparation that are out of their control, professionals working in frontline units, resort in a rather disengaged attitude, which consequently has a negative impact on their psychological health. This is in line with the goodness of fit hypothesis proposed by Lazarus and Folkman (Lazarus & Folkman, 1984), which assumes that emotion‐focused coping (e.g., denial) is preferred in uncontrollable situations as opposed to problem‐focused coping (e.g., problem‐solving) in controllable situations.

In the present study, two of the most common and strongest predictors of poorer QoL were depression and occupational burnout, which is in accordance with previous studies (Stojanov et al., 2020; Suryavanshi et al., 2020). Depression and occupational burnout may make healthcare professionals more vulnerable to experiencing poorer QoL in all aspects of their lives, while depressive mood may also encourage a general negative perception in these outcomes (Berlim & Fleck, 2007). Longitudinal studies will be invaluable in deciphering whether QoL challenges in healthcare professionals persist in the longer term, due to depression and occupational burnout during the pandemic.

### 5.1 Limitations

The cross‐sectional nature of the study means there are inherent limitations in maintaining causality in the observed relationships. While the electronic distribution of the questionnaires and anonymity maximised recruitment and completion rates, they limited the collection of longitudinal data on the progression of psychosocial challenges in the same sample. Participants were recruited through multiple means. This, along with the anonymous participation, may have inadvertently led to the possibility of duplicate entries by some participants. This limitation was partially addressed by following a complete case analysis, which helped exclude duplicate cases where the survey was incomplete in the first attempt.

### 5.2 Generalisability and transferability

Much of the sample consisted of nurses and midwives while other groups were comparatively under‐represented. Caution is warranted into the generalisability of the study findings to the wider population of healthcare professionals in Cyprus, especially doctors and allied health professionals. However, the large sample size of nurses and midwives facilitates generalisability to that population. Furthermore, the findings are consistent with the literature in other contexts and settings, including various countries and during different time periods of the COVID‐19 pandemic, supporting their transferability.

### 5.3 Implications for clinical practice

Despite these limitations, this study has important implications for future research, clinical practice, and public health. It uniquely captured the impact of the pandemic in a period of relative stability in terms of new infections in Cyprus (May‐October) suggesting that psychosocial difficulties in healthcare professionals persist even after the initial surge observed worldwide during March‐April 2020. It is also a large study that sought to identify risk factors that could help inform immediate public health initiatives and psychological interventions to help healthcare professionals build resilience against the inevitable psychosocial burden of the COVID‐19 pandemic. Emergency psychological interventions may be in the form of online mental health services, which have the potential to ameliorate some of the stress in healthcare professionals during this pandemic (Liu et al., 2020). In Cyprus, psychological support services are currently available only for people diagnosed with COVID‐19 with a history of mental health challenges and their relatives in the form of video and telephone sessions and for the whole population in the form of a telephone helpline (Press & Information Office, 2020b), whereas there are no specific services directed at healthcare professionals and their needs. The findings can also inform the development of long‐term action plans and preparedness protocols to include training in the use of adaptive coping strategies during future outbreaks and public health emergencies. Such initiatives need to account for the limitations in the utilisation of effective coping strategies by healthcare professionals in emergency situations. For instance, utilising social support, which is generally considered an adaptive coping mechanism, may need to be re‐invented within the context of social distancing and isolation of a pandemic situation. On the other hand, healthcare professionals may be trained towards adopting more approach rather than avoidance coping strategies, which may be particularly challenging in an uncontrollable situation like the COVID‐19 pandemic. Any successful public health endeavour will likely entail a complex interplay of personal, social, organisational, and economic factors. Our study identified several of the factors that may be considered in a well‐informed public health approach.

## 6 CONCLUSIONS

The findings of the present study highlight the psychosocial consequences of the COVID‐19 pandemic in healthcare professionals with observed notable levels of anxiety and depression. We have identified several risk factors for poor QoL, anxiety, depression, and occupational burnout, including female gender, medical profession (vs non‐medical), frontline units (inpatient wards, ICUs), inadequate workplace preparation to deal with the pandemic, and engaging in avoidance coping. The presence of depression and anxiety further impedes QoL in healthcare professionals. These findings can inform immediate psychological interventions, public health initiatives and future protocols to help healthcare professionals cope with the psychosocial burden of COVID‐19 and similar health crises in the future.

## CONFLICTS OF INTEREST

None declared.

## Supporting information

Supplementary Material

Supplementary Material

Supplementary Material

Supplementary Material

Supplementary Material

## Data Availability

The data that support the findings of this study are available from the corresponding author upon reasonable request.
