# Supplementary material for: Beyond the physical risk: Psychosocial impact and coping in healthcare professionals during the COVID‐19 pandemic
Source: J Clin Nurs. 2021 Jul 6;34(12):5234–48. doi: 10.1111/jocn.15938 (PMC8447326; doi:10.1111/jocn.15938)
Supplement: Supplementary file 2 — Supplementary Material [file JOCN-34-5234-s001.docx]

**Missing Value Approach**

1. **Valid/eligible records**

**
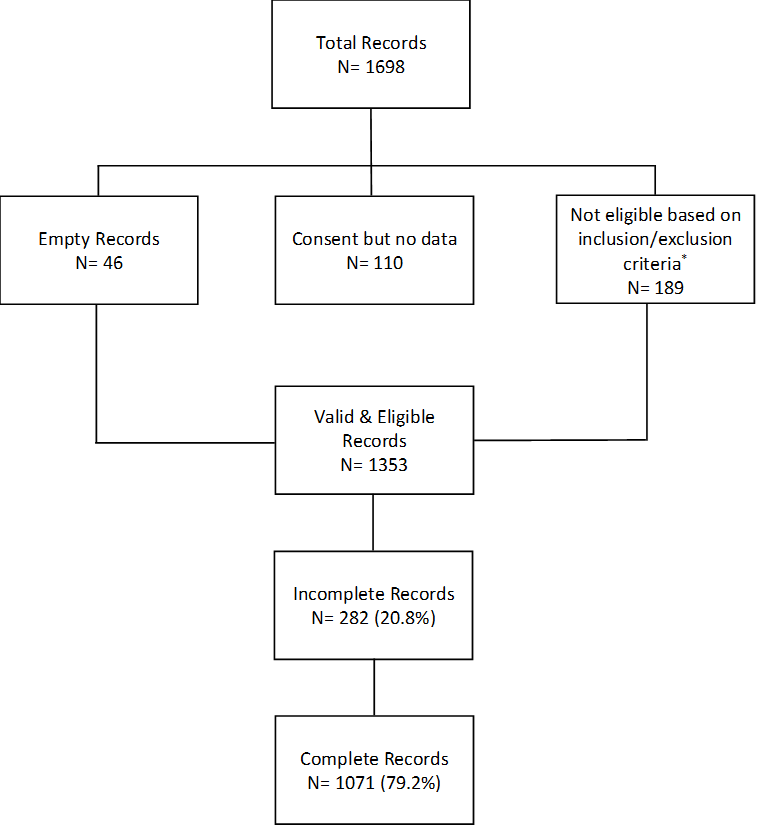
**

^*^did not fall under the definition of healthcare professionals, or were not active at the time, or were not living in Cyprus

1. **Missing value analysis**

**(total cases: n=1353, complete cases: n=1071, incomplete cases: n=282)**

|  | ***Variables*** | ***Missing values (%)*** |
| --- | --- | --- |
| DEMOGRAPHICS | Age | 14 (1) |
|  | Gender | 0 |
|  | Family status | 4 (0.3) |
|  | Education level | 4 (0.3) |
|  | Profession | 2 (0.1) |
|  | Years of experience | 40 (3) |
|  | Healthcare setting | 6 (0.4) |
|  | Crisis experience | 5 (0.4) |
|  | Work during COVID-19 | 0 |
|  | Frontline | 6 (0.4) |
|  | Workplace preparation | 7 (0.5) |
|  | Self-isolation | 2 (0.1) |
|  | COVID-19 diagnosis | 8 (0.6) |
|  | **Total values: n= 17589** | **Missing values: *n=* 98 (0.6%)** |
| PSYCHOSOCIAL | *WHOQOL-BREF* |  |
|  | Overall QoL/Health | 139 (10.3) |
|  | Physical health | 140 (10.3) |
|  | Psychological health | 145 (10.7) |
|  | Social relationships | 142 (10.5) |
|  | Environment | 139 (10.3) |
|  | *GAD-7* | 193 (14.3) |
|  | *PHQ-8* | 194 (14.3) |
|  | *CBI* | 216 (16) |
|  | *BRIEF COPE* |  |
|  | Self-distraction | 243 (18) |
|  | Active coping | 243 (18) |
|  | Denial | 243 (18) |
|  | Substance use | 243 (18) |
|  | Emotional support | 243 (18) |
|  | Instrumental support | 243 (18) |
|  | Venting | 243 (18) |
|  | Positive reframing | 243 (18) |
|  | Planning | 243 (18) |
|  | Humour | 243 (18) |
|  | Acceptance | 243 (18) |
|  | Religion | 243 (18) |
|  | Self-blame | 243 (18) |
|  | Behavioural disengagement | 243 (18) |
|  | **Total values: n= 29766** | **Missing values: *n=* 4709 (15.8%)** |

Little’s MCAR test was significant (Chi-square= 160.42, *df*= 109, *p*= .001).

1. **Demographic differences between participants with complete vs incomplete data**

| ***Demographic Variable*** | ***All Cases*** | ***Complete Cases*** | ***Incomplete Cases*** | ***Test Statistic*** | ***Sig.*** |
| --- | --- | --- | --- | --- | --- |
| Age (mean, SD) | 36.81 (9.20) | 36.86 (8.93) | 36.57 (10.25) | *t*(374.58)= -.43 | .67 |
|  | n= 1339 | n= 1071 | n= 268 |  |  |
| Gender (n, %) |  |  |  | χ^2^(1)= .14 | .71 |
| Female | 991 (73.2) | 782 (73) | 209 (74.1) |  |  |
| Male | 362 (26.8) | 289 (27) | 73 (25.9) |  |  |
|  | n= 1353 | n= 1071 | n= 282 |  |  |
| Family status (n, %) |  |  |  | χ^2^(1)= .97 | .32 |
| Married/relationship | 978 (72.5) | 783 (73.1) | 195 (70.1) |  |  |
| Single | 371 (27.5) | 288 (26.9) | 83 (29.9) |  |  |
|  | n= 1349 | n= 1071 | n= 278 |  |  |
| Education level (n, %) |  |  |  | χ^2^(1)= 19.50 | <.001 |
| School | 25 (1.9) | 11 (1) | 14 (5) |  |  |
| University | 1324 (98.1) | 1060 (99) | 264 (95) |  |  |
|  | n= 1349 | n= 1071 | n= 278 |  |  |
| Profession (n, %) |  |  |  | χ^2^(4)= 2.42 | .66 |
| Doctors | 49 (3.6) | 39 (3.6) | 10 (3.6) |  |  |
| Nurses & midwives | 1223 (90.5) | 974 (90.9) | 249 (88.9) |  |  |
| Allied health professionals | 48 (3.6) | 36 (3.4) | 12(4.3) |  |  |
| Administrative & academic | 13 (1) | 10 (0.9) | 3 (1.1) |  |  |
| Support | 18 (1.3) | 12 (1.1) | 6 (2.1) |  |  |
|  | n= 1351 | n= 1071 | n= 280 |  |  |
| Years of experience (mean, SD) | 13.31 (8.62) | 13.30 (8.47) | 13.33 (9.28) | *t*(337.55)= .05 | .96 |
|  | n= 1313 | n=1071 | n=242 |  |  |

**(*continued)***

| ***Demographic Variable*** | ***All Cases*** | ***Complete Cases*** | ***Incomplete Cases*** | ***Test Statistic*** | ***Sig.*** |
| --- | --- | --- | --- | --- | --- |
| Healthcare setting (n, %) |  |  |  | χ^2^(8)= 12.68 | .12 |
| Primary | 104 (7.7) | 80 (7.5) | 24 (8.7) |  |  |
| Outpatient | 60 (4.5) | 47 (4.4) | 13 (4.7) |  |  |
| Emergency | 139 (10.3) | 111 (10.4) | 28 (10.1) |  |  |
| Inpatient | 622 (46.2) | 511 (47.7) | 111 (40.2) |  |  |
| Intensive care unit | 171 (12.7) | 138 (12.9) | 33 (12) |  |  |
| Mental health | 53 (3.9) | 41 (3.8) | 12 (4.3) |  |  |
| Specialised | 84 (6.2) | 60 (5.6) | 24 (8.7) |  |  |
| Lab | 42 (3.1) | 34 (3.2) | 8 (2.9) |  |  |
| Public health | 72 (5.3) | 49 (4.6) | 23 (8.3) |  |  |
|  | n= 1347 | n= 1071 | n= 276 |  |  |
| Healthcare crisis experience (n, %) |  |  |  | χ^2^(1)= .00 | .95 |
| Yes | 211 (15.7) | 168 (15.7) | 43 (15.5) |  |  |
| No | 1137 (84.3) | 903 (84.3) | 234 (84.5) |  |  |
|  | n= 1348 | n= 1071 | n= 277 |  |  |
| Frontline (n, %) |  |  |  | χ^2^(1)= 5.04 | .03 |
| Yes | 593 (44) | 488 (45.6) | 205 (38) |  |  |
| No | 754 (56) | 583 (54.4) | 171 (62) |  |  |
|  | n= 1347 | n= 1071 | n= 276 |  |  |
| COVID-19 preparation (n, %) |  |  |  | χ^2^(1)= .14 | .71 |
| Yes | 765 (56.8) | 606 (56.6) | 159 (57.8) |  |  |
| No | 581 (43.2) | 465 (43.4) | 116 (42.2) |  |  |
|  | n= 1346 | n= 1071 | n= 275 |  |  |

**(*continued)***

| ***Demographic Variable*** | ***All Cases*** | ***Complete Cases*** | ***Incomplete Cases*** | ***Test Statistic*** | ***Sig.*** |
| --- | --- | --- | --- | --- | --- |
| Self-isolation (n, %) |  |  |  | χ^2^(1)= .66 | .42 |
| Yes | 457 (33.8) | 368 (34.4) | 89 (31.8) |  |  |
| No | 894 (66.2) | 703 (65.6) | 191 (68.2) |  |  |
|  | n= 1351 | n= 1071 | n= 280 |  |  |
| COVID-19 diagnosis (n, %) |  |  |  | χ^2^(1)= .79 | .37 |
| Yes | 29 (2.2) | 25 (2.3) | 4 (1.5) |  |  |
| No | 1316 (97.8i) | 1046 (97.7) | 270 (98.5) |  |  |
|  | n= 1345 | n= 1071 | n= 274 |  |  |

1. **Psychosocial differences between participants with complete vs incomplete data**

| ***Psychosocial Variable (mean, SD)*** | ***All Cases*** | ***Complete Cases*** | ***Incomplete Cases*** | ***Test Statistic*** | ***Sig.*** |
| --- | --- | --- | --- | --- | --- |
| Overall QoL/health | 15.85 (2.49) | 15.87 (2.46) | 15.71 (2.73) | *t*(1212)= -.75 | .45 |
|  | n= 1214 | n= 1071 | n= 143 |  |  |
| Physical health | 14.94 (2.04) | 14.93 (2.04) | 15 (2.04) | *t*(1211)= .36 | .72 |
|  | n= 1213 | n= 1071 | n= 142 |  |  |
| Psychological health | 14.93 (2.22) | 14.92 (2.22) | 14.97 (2.24) | *t*(1206)= .23 | .82 |
|  | n= 1208 | n= 1071 | n= 137 |  |  |
| Social relationships | 15.04 (2.41) | 15 (2.41) | 15.32 (2.35) | *t*(1209)= 1.50 | .14 |
|  | n= 1211 | n= 1071 | n= 140 |  |  |
| Environment | 14.22 (1.87) | 14.21 (1.86) | 14.27 (1.98) | *t*(1212)= .38 | .71 |
|  | n= 1214 | n= 1071 | n= 143 |  |  |
| Occupational burnout | 343.54 (150.98) | 346.08 (149.92) | 302.27 (163.01) | *t*(1135)= -2.29 | .02 |
|  | n= 1137 | n= 1071 | n= 66 |  |  |
| Anxiety | 7.42 (4.66) | 7.42 (4.63) | 7.48 (5.02) | *t*(1158)= .13 | .89 |
|  | n= 1160 | n= 1071 | n= 89 |  |  |
| Depression | 7.29 (4.88) | 7.26 (4.80) | 7.63 (5.83) | *t*(96.93)= .57 | .57 |
|  | n= 1159 | n= 1071 | n= 88 |  |  |
| Self-distraction | 2.36 (0.77) | 2.36 (0.77) | 2.37 (0.78) | *t*(1108)= .09 | .93 |
|  | n= 1110 | n= 1071 | n= 39 |  |  |
| Active coping | 2.73 (0.81) | 2.73 (0.81) | 2.69 (0.82) | *t*(1108)= -.31 | .75 |
|  | n= 1110 | n= 1071 | n= 39 |  |  |
| Denial | 1.80 (0.72) | 1.79 (0.72) | 1.88 (0.88) | *t*(1108)= .78 | .44 |
|  | n= 1110 | n= 1071 | n= 39 |  |  |
| Substance use | 1.12 (0.42) | 1.11 (0.42) | 1.17 (0.45) | *t*(1108)= .75 | .45 |
|  | n= 1110 | n= 1071 | n= 39 |  |  |

***(continued)***

| ***Demographic Variable (mean, SD)*** | ***All Cases*** | ***Complete Cases*** | ***Incomplete Cases*** | ***Test Statistic*** | ***Sig.*** |
| --- | --- | --- | --- | --- | --- |
| Emotional support | 2.21 (0.83) | 2.21 (0.83) | 2.33 (0.98) | *t*(40.02)= .79 | .44 |
|  | n= 1110 | n= 1071 | n= 39 |  |  |
| Instrumental support | 2.31 (0.82) | 2.30 (0.82) | 2.47 (0.87) | *t*(1108)= 1.27 | .20 |
|  | n= 1110 | n= 1071 | n= 39 |  |  |
| Venting | 2.41 (0.73) | 2.40 (0.73) | 2.56 (0.76) | *t*(1108)= 1.35 | .18 |
|  | n= 1110 | n= 1071 | n= 39 |  |  |
| Positive reframing | 3.05 (0.79) | 3.05 (0.79) | 3.03 (0.86) | *t*(1108)= -.18 | .86 |
|  | n= 1110 | n= 1071 | n= 39 |  |  |
| Planning | 3.04 (0.75) | 3.04 (0.76) | 3 (0.71) | *t*(1108)= -.32 | .75 |
|  | n= 1110 | n= 1071 | n= 39 |  |  |
| Humour | 2.17 (0.75) | 2.18 (0.75) | 2.08 (0.75) | *t*(1108)= -.82 | .42 |
|  | n= 1110 | n= 1071 | n= 39 |  |  |
| Acceptance | 2.88 (0.72) | 2.88 (0.72) | 2.94 (0.77) | *t*(1108)= .49 | .62 |
|  | n= 1110 | n= 1071 | n= 39 |  |  |
| Religion | 2.32 (0.99) | 2.31 (1) | 2.60 (0.93) | *t*(1108)= 1.81 | .07 |
|  | n= 1110 | n= 1071 | n= 39 |  |  |
| Self-blame | 2.48 (0.73) | 2.49 (0.73) | 2.29 (0.75) | *t*(1108)= 1.60 | .11 |
|  | n= 1110 | n= 1071 | n= 39 |  |  |
| Behavioural disengagement | 1.39 (0.60) | 1.39 (0.60) | 1.40 (0.61) | *t*(1108)= .04 | .97 |
|  | n= 1110 | n= 1071 | n= 39 |  |  |
