# Supplementary figures and images for: Beyond the physical risk: Psychosocial impact and coping in healthcare professionals during the COVID‐19 pandemic
Source: J Clin Nurs. 2021 Jul 6;34(12):5234–48. doi: 10.1111/jocn.15938 (PMC8447326; doi:10.1111/jocn.15938)

**Scree Plot from the Exploratory Factor Analysis on the Brief COPE**


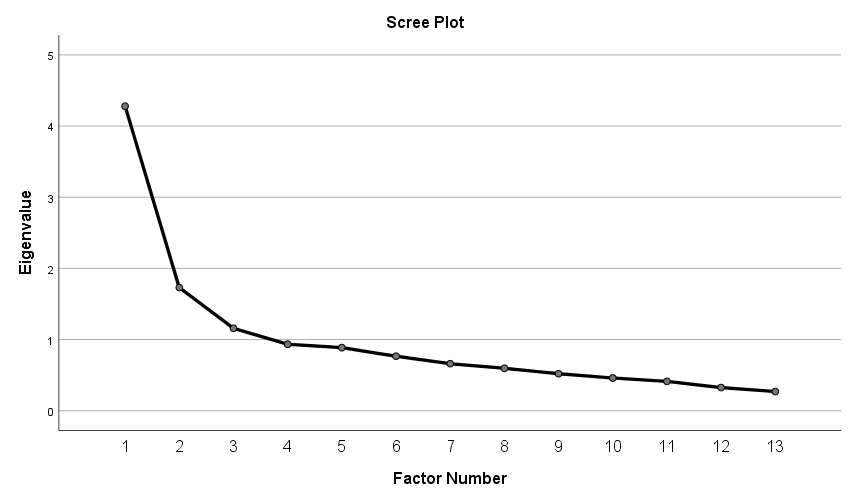

Supplement: Supplementary file 3 — Supplementary Material [file JOCN-34-5234-s003.docx]
