# Supplementary material for: Beyond the physical risk: Psychosocial impact and coping in healthcare professionals during the COVID‐19 pandemic
Source: J Clin Nurs. 2021 Jul 6;34(12):5234–48. doi: 10.1111/jocn.15938 (PMC8447326; doi:10.1111/jocn.15938)
Supplement: Supplementary file 4 — Supplementary Material [file JOCN-34-5234-s004.docx]

**Correlations between the study variables**

| Variable | 1 | 2 | 3 | 4 | 5 | 6 | 7 | 8 | 9 | 10 | 11 | 12 | 13 | 14 | 15 | 16 | 17 |
| --- | --- | --- | --- | --- | --- | --- | --- | --- | --- | --- | --- | --- | --- | --- | --- | --- | --- |
| 1. Overall QoL/ health | - |  |  |  |  |  |  |  |  |  |  |  |  |  |  |  |  |
| 2. Physical health | .53^**^ | - |  |  |  |  |  |  |  |  |  |  |  |  |  |  |  |
| 3. Psychological health | .55^**^ | .72^**^ | - |  |  |  |  |  |  |  |  |  |  |  |  |  |  |
| 4. Social relationships | .44^**^ | .62^**^ | .68^**^ | - |  |  |  |  |  |  |  |  |  |  |  |  |  |
| 5. Environment | .42^**^ | .64^**^ | .55^**^ | .56^**^ | - |  |  |  |  |  |  |  |  |  |  |  |  |
| 6. Anxiety | -.31^**^ | -.54^**^ | -.55^**^ | -.48^**^ | -.40^**^ | - |  |  |  |  |  |  |  |  |  |  |  |
| 7. Depression | -.40^**^ | -.65^**^ | -.66^**^ | -.58^**^ | -.45^**^ | .77^**^ | - |  |  |  |  |  |  |  |  |  |  |
| 8. Occupational burnout | -.31^**^ | -.50^**^ | -.41^**^ | -.40^**^ | -.40^**^ | .53^**^ | .53^**^ | - |  |  |  |  |  |  |  |  |  |
| 9. Approach coping | -.06^*^ | .03 | .03 | .02 | .06 | .07^*^ | .05 | -.01 | - |  |  |  |  |  |  |  |  |
| 10. Support-seeking coping | -.08^*^ | -.06^*^ | -.08^*^ | .03 | .01 | .18^**^ | .12^**^ | .06^*^ | .49^**^ | - |  |  |  |  |  |  |  |
| 11. Avoidance coping | -.25^**^ | -.32^**^ | -.43^**^ | -.34^**^ | -.21^**^ | .46^**^ | .48^**^ | .29^**^ | .15^**^ | .26^**^ | - |  |  |  |  |  |  |
| 12. Gender | -.03 | -.08^*^ | -.11^**^ | -.02 | -.04 | .10^**^ | .06 | .07^*^ | .09^*^ | .14^**^ | .04 | - |  |  |  |  |  |
| 13. Age | -.14^**^ | -.00 | -.06 | -.06 | -.06 | -.06 | -.04 | -.02 | .18^**^ | -.00 | .12^**^ | .02 | - |  |  |  |  |
| 14. Family status | -.01 | -.03 | .01 | .13^**^ | -.05 | .06^*^ | -.00 | .06 | -.00 | .04 | .01 | .02 | .14^**^ | - |  |  |  |
| 15. Education level | .02 | .05 | .04 | .04 | .04 | -.02 | -.01 | -.01 | -.01 | -.00 | -.04 | -.02 | -.14^**^ | .02 | - |  |  |
| 16. Doctors | .05 | .03 | -.03 | -.06^*^ | .02 | -.02 | -.04 | -.04 | .03 | .01 | -.03 | -.03 | .12^**^ | -.01 | -.03 | - |  |
| 17. Nurses & Midwives | -.02 | -.06^*^ | -.00 | .01 | -.05 | .06 | .07^*^ | .10^**^ | -.07^*^ | -.03 | .02 | .01 | -.19^**^ | .01 | .29^**^ | -.62^**^ | - |

^*^*p*≤ .05, ^**^*p*≤ .01

**(*continued)***

| Variable | 1 | 2 | 3 | 4 | 5 | 6 | 7 | 8 | 9 | 10 | 11 | 12 | 13 | 14 | 15 | 16 | 17 |
| --- | --- | --- | --- | --- | --- | --- | --- | --- | --- | --- | --- | --- | --- | --- | --- | --- | --- |
| 18. Years of experience | -.11^**^ | -.01 | -.04 | -.05 | -.04 | -.05 | -.04 | .00 | .16^**^ | .01 | .10^**^ | .06^*^ | .92^**^ | .16^**^ | -.12^**^ | .02 | -.06 |
| 19. Crisis experience | -.04 | .00 | .00 | -.01 | -.01 | -.00 | -.01 | -.02 | .03 | -.02 | .01 | -.04 | .25^**^ | .01 | -.03 | -.00 | -.04 |
| 20. Primary setting | .08^*^ | .05 | .05 | -.01 | .01 | -.05 | -.04 | -.19^**^ | -.02 | -.05 | -.11^**^ | .04 | -.04 | -.04 | -.01 | .15^**^ | -.13^**^ |
| 21. Outpatient setting | -.03 | .02 | .03 | -.02 | -.03 | -.05 | -.04 | -.05 | .04 | -.02 | .04 | .05 | .15^**^ | -.00 | -.02 | .15^**^ | -.09^*^ |
| 22. Emergency setting | -.01 | .03 | .03 | .01 | .01 | -.02 | -.04 | .01 | -.03 | -.11^**^ | -.02 | -.13^**^ | -.03 | -.04 | .00 | -.02 | .01 |
| 23. Inpatient setting | .01 | -.07^*^ | -.04 | .05 | -.03 | .07^*^ | .05 | .13^**^ | -.01 | .07^*^ | .01 | .10^**^ | -.06 | .05 | .02 | -.05 | .11^**^ |
| 24. ICU setting | -.00 | .01 | .02 | -.04 | .03 | .02 | .03 | .07^*^ | -.01 | -.01 | .04 | -.04 | -.02 | -.02 | -.04 | -.06 | .05 |
| 25. Mental health setting | -.03 | .02 | .01 | -.03 | .04 | -.10^*^ | -.08^*^ | -.02 | .01 | .06^*^ | -.03 | -.13^**^ | .02 | -.05 | .02 | -.01 | .03 |
| 26. Specialised setting | -.00 | -.01 | .01 | -.01 | -.04 | -.01 | .02 | -.03 | .02 | -.01 | -.02 | -.02 | -.05 | -.04 | .03 | -.03 | -.05 |
| 27. Lab setting | -.03 | -.01 | -.05 | -.01 | -.03 | .01 | .03 | -.01 | -.04 | -.04 | .04 | .00 | .04 | .05 | -.03 | -.04 | -.07^*^ |
| 28. Frontline | -.03 | -.05 | -.01 | -.03 | -.03 | .06 | .05 | .11^**^ | -.02 | -.01 | .00 | -.09^*^ | .03 | .03 | .00 | .00 | .02 |
| 29. Preparation | .13^**^ | .15^**^ | .11^**^ | .13^**^ | .18^**^ | -.10^**^ | -.09^*^ | -.26^**^ | -.06 | -.05 | -.08^*^ | -.02 | -.01 | -.05 | -.01 | .07^*^ | -.07^*^ |
| 30. Self-isolation | -.03 | -.01 | -.01 | -.04 | -.01 | .04 | .03 | .07^*^ | .00 | -.01 | -.01 | .02 | -.01 | -.01 | -.00 | -.05 | .02 |
| 31. COVID-19 diagnosis | -.05 | -.00 | .01 | -.03 | -.07^*^ | .00 | .00 | .05 | -.02 | -.04 | .06^*^ | -.03 | .00 | .02 | .02 | .00 | .01 |

^*^*p*≤ .05, ^**^*p*≤ .01

**(*continued)***

| Variable | 18 | 19 | 20 | 21 | 22 | 23 | 24 | 25 | 26 | 27 | 28 | 29 | 30 | 31 |
| --- | --- | --- | --- | --- | --- | --- | --- | --- | --- | --- | --- | --- | --- | --- |
| 18. Years of experience | - |  |  |  |  |  |  |  |  |  |  |  |  |  |
| 19. Crisis experience | .25^**^ | - |  |  |  |  |  |  |  |  |  |  |  |  |
| 20. Primary setting | -.06 | .03 | - |  |  |  |  |  |  |  |  |  |  |  |
| 21. Outpatient setting | .13^**^ | .03 | -.06^*^ | - |  |  |  |  |  |  |  |  |  |  |
| 22. Emergency setting | -.05 | .06 | -.10^*^ | -.07^*^ | - |  |  |  |  |  |  |  |  |  |
| 23. Inpatient setting | -.01 | -.07^*^ | -.27^**^ | -.21^**^ | -.33^**^ | - |  |  |  |  |  |  |  |  |
| 24. ICU setting | .01 | .02 | -.11^**^ | -.08^*^ | -.13^**^ | -.37^**^ | - |  |  |  |  |  |  |  |
| 25. Mental health setting | .03 | -.02 | -.06 | -.04 | -.07^*^ | -.19^**^ | -.08^*^ | - |  |  |  |  |  |  |
| 26. Specialised setting | -.08^*^ | -.02 | -.07^*^ | -.05 | -.08^*^ | -.23^**^ | -.09^*^ | -.05 | - |  |  |  |  |  |
| 27. Lab setting | .02 | -.01 | -.05 | -.04 | -.06^*^ | -.17^**^ | -.07^*^ | -.04 | -.04 | - |  |  |  |  |
| 28. Frontline | .05 | .09^*^ | -.10^*^ | -.10^**^ | .18^**^ | -.04 | .25^**^ | -.12^**^ | -.12^**^ | .01 | - |  |  |  |
| 29. Preparation | -.03 | .03 | .06^*^ | .02 | -.02 | -.12^**^ | .05 | -.04 | .05 | .08^*^ | -.05 | - |  |  |
| 30. Self-isolation | .01 | .04 | -.12^**^ | -.06 | -.00 | .02 | .16^**^ | -.03 | -.07^*^ | .00 | .23^**^ | -.06 | - |  |
| 31. COVID-19 diagnosis | .01 | .02 | -.04 | -.03 | .01 | .05 | .05 | -.03 | -.04 | -.03 | .12^**^ | -.09^*^ | .18^**^ | - |

^*^*p*≤ .05, ^**^*p*≤ .01
