# Supplementary material for: Beyond the physical risk: Psychosocial impact and coping in healthcare professionals during the COVID‐19 pandemic
Source: J Clin Nurs. 2021 Jul 6;34(12):5234–48. doi: 10.1111/jocn.15938 (PMC8447326; doi:10.1111/jocn.15938)
Supplement: Supplementary file 5 — Supplementary Material [file JOCN-34-5234-s002.docx]

**Summary of Significant Predictors of Psychosocial Outcomes in Multiple Regression Analyses**

|  | Overall QoL/ Health | Physical Health | Psychological Health | Social Relationships | Environment | Anxiety | Depression | Occupational burnout |
| --- | --- | --- | --- | --- | --- | --- | --- | --- |
| Gender^†^ |  |  | ▼ |  |  | ▲ |  | ▲ |
| Age | ▼ |  |  |  |  |  |  |  |
| Family status^‡^ |  |  |  | ▲ |  |  |  |  |
| Doctors^§^ |  |  |  | ▼ |  |  |  | ▲ |
| Nurses & midwives^§^ |  |  |  |  |  |  | ▲ | ▲ |
| Inpatient setting^¶^ |  |  |  |  |  |  |  | ▲ |
| ICU setting^¶^ |  |  |  |  |  |  |  | ▲ |
| Mental health setting^¶^ |  |  |  |  |  | ▼ |  |  |
| Frontline^¥^ |  |  |  |  |  |  |  | ▲ |
| Preparation^¥^ | ▲ |  |  | ▲ | ▲ | ▼ | ▼ | ▼ |
| COVID-19 diagnosis^¥^ |  |  |  |  | ▼ |  |  |  |
| Anxiety |  |  |  |  | ▼ | - | - | - |
| Depression | ▼ | ▼ | ▼ | ▼ | ▼ | - | - | - |
| Occupational burnout | ▼ | ▼ |  | ▼ | ▼ | - | - | - |
| Avoidance coping |  |  | ▼ | ▼ |  | ▲ | ▲ | ▲ |

*Note:* - indicates that the variable was not part of the multiple regression model; ▲Significant positive relationship, ▼Significant negative relationship

^†^Male=1, female=2, ^‡^Single=0, Married/in a relationship=1, ^§^Dummy coded: reference group was non-medical profession, ^¶^Dummy-coded: reference group was public health setting, ^¥^No= 0, Yes= 1
